# Causal Associations of Cerebrospinal Fluid and Circulating Metabolites With Malignant Brain Neoplasms: A Mendelian Randomization Analysis

**DOI:** 10.1002/brb3.71319

**Published:** 2026-03-31

**Authors:** Xinyin Zhang, Zhicheng Wu, Jing Tan, Mengting Hu, Feixia Pan, Ting Tao

**Affiliations:** ^1^ National Clinical Research Center for Children and Adolescents' Health and Diseases Children's Hospital Zhejiang University School of Medicine Hangzhou China; ^2^ Zhejiang Key Laboratory of Neonatal Diseases Hangzhou China; ^3^ Department of Environmental Health and Engineering Johns Hopkins University Baltimore Maryland USA; ^4^ Department of Clinical Laboratory Children's Hospital Zhejiang University School of Medicine Hangzhou China; ^5^ Department of Surgical Oncology Children's Hospital Zhejiang University School of Medicine, National Clinical Research Center for Children and Adolescents' Health and Diseases Hangzhou China; ^6^ Cancer Center Zhejiang University Hangzhou China

**Keywords:** brain tumor, cerebrospinal fluid, genome‐wide association study, Mendelian randomization, metabolites

## Abstract

**Background:**

Emerging insights from immunometabolism underscore the importance of metabolic–immune interactions in shaping the brain tumor microenvironment and potentially influencing brain behavior and neurological outcomes. Although previous studies have suggested potential links between metabolites and risks of malignant brain tumor development, the causal relationship remains unclarified.

**Methods:**

Exposures were extracted from 136,016 Europeans for 233 circulating metabolites and from 689 participants for 338 cerebrospinal fluid (CSF) metabolite levels. Genome‐wide association study (GWAS) data for malignant neoplasm of the brain (NCase = 1070, NControl = 345,118) from the FinnGen consortium were used. The primary univariable Mendelian randomization (UVMR) analysis used inverse variance weighting (IVW) and three additional methods for causal assessment. Multivariable MR (MVMR) assessed the direct effects of metabolites, and suggestive biomarkers underwent metabolic pathway enrichment.

**Results:**

Twelve metabolic pathways and 21 metabolites (4 circulating biomarkers and 17 CSF metabolites) potentially linked to brain malignancy. The strongest associations were observed for the ratio of omega‐3 fatty acids to total fatty acids (IVW: *p* = 0.01, OR: 0.74, 95% CI: 0.58–0.94) and 5‐methylthioadenosine (IVW: *p* = 0.001, OR: 1.25, 95% CI: 1.09–1.44). Sensitivity analyses confirmed result robustness. MVMR analysis demonstrated direct effects of blood albumin, along with CSF metabolites such as alpha‐hydroxyisocaproate, 1‐ribosyl‐imidazoleacetate, 5‐methylthioadenosine, N1‐methylinosine, and urate, independent of other factors.

**Conclusions:**

This study reveals metabolite–brain tumor links, shows biomarker potential for screening and prevention, and offers new oncology insights for mechanisms and therapies.

## Graphical abstract

This study systematically examined circulating and cerebrospinal fluid metabolites in relation to malignant brain neoplasms. Leveraging large‐scale population‐based genome‐wide association data, the integrated analysis identified metabolites and metabolic pathways potentially linked to brain malignancy, providing supportive evidence for biomarker exploration.

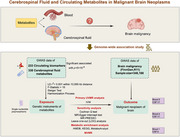

**Abbreviations**
CNS: central nervous system
CSF: cerebrospinal fluid
GBM: glioblastoma multiforme
GWAS: genome‐wide association study
IVs: instrumental variables
IVW: inverse variance weighting
LD: linkage disequilibrium
MR‐Egger: MR‐Egger regression
MR‐PRESSO: MR Pleiotropy RESidual Sum and Outlier
MTA: 5‐Methylthioadenosine
MVMR: multivariable MR
MWAS: metabolome‐wide association study
SNPs: single nucleotide polymorphisms
UVMR: univariable Mendelian randomization

## 1 Introduction

Malignant neoplasm of the brain, whether primary or metastatic, is among the most aggressive and deadly forms of cancer, spreading rapidly within the brain and spine. Despite their relative rarity, brain malignancies have a significant impact on global health, with an estimated 321,476 new cases and 248,305 deaths worldwide in 2022, representing 1.6% of all cancer diagnoses but 2.6% of cancer‐related deaths. These tumors, particularly those in the cerebrum, cause diverse and often debilitating symptoms depending on their location (Bray et al. 2024). Advances in treatment have done little to improve outcomes for many patients, as malignant brain and central nervous system (CNS) tumors have a 5‐year survival rate of only 35.7%, and the prognosis is even grimmer for aggressive subtypes like glioblastoma multiforme (GBM), with survival rates below 5% (El Kheir et al. 2022). These stark figures underscore the urgent need for novel strategies in prevention, early detection, and risk stratification to improve patient outcomes.

Metabolomics, the large‐scale study of small molecules, has become an essential tool in unraveling the metabolic underpinnings of tumorigenesis and identifying biomarkers critical for diagnosis and treatment (Schmidt et al. 2021). Metabolic profiling of blood and cerebrospinal fluid (CSF), including metabolites such as nucleotides, amino acids, and carbohydrates, offers valuable insights into the biochemical pathways driving tumorigenesis in the brain. Recent research has highlighted specific blood metabolites associated with brain malignancies, including elevated levels of α‐ and γ‐tocopherol in glioblastoma (Björkblom et al. 2016), as well as altered levels of lactate, fumarate, and steroid hormone metabolites (Löding et al. 2024). Although less frequently studied, CSF metabolites present an even more direct window into brain cancer biology due to their proximity to the CNS. Key findings include 2‐hydroxyglutarate as a biomarker in IDH‐mutant gliomas and the role of succinate and lactic acid in brain tumor diagnosis and monitoring (Fujita et al. 2022). These metabolic signatures underscore the potential of metabolomics to uncover novel therapeutic targets and improve the management of brain malignancies.

Investigating CSF and blood metabolites can directly mirror the nutritional and metabolic status of the CNS microenvironment, with their abnormalities serving as critical links connecting dietary patterns, metabolic dysfunction, and the development of brain malignancies (Liu et al. 2025). Conventional observational studies, however, are limited by confounding and reverse causality, and randomized controlled trials are often impractical in metabolomics research (Shang et al. 2025). To address these challenges, we used Mendelian randomization (MR), an epidemiological approach that leverages genetic variants as proxies for exposures to assess causal links between exposure and disease, minimizing bias (Huang et al. 2025). We applied a comprehensive MR approach to evaluate genetic associations of 233 circulating metabolic biomarkers and 338 CSF metabolites with brain malignancy risk. This approach aims to identify biomarkers for early diagnosis and provide insights into brain tumor biology and prevention strategies.

## 2 Materials and Methods

### 2.1 Study Design and Procedure

**FIGURE 1:**
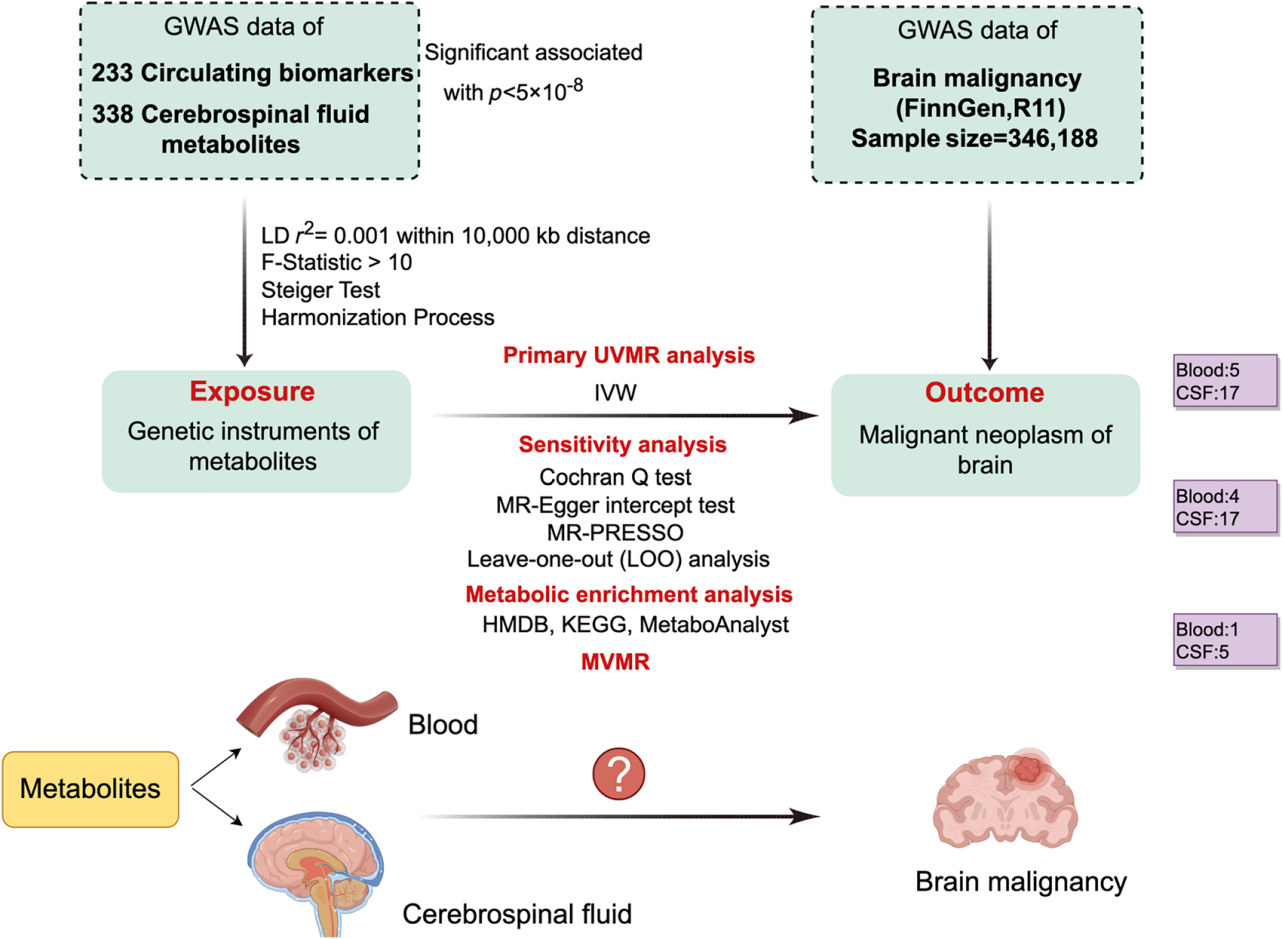
Overview of the Mendelian randomization (MR) analysis pipeline used in this study. IVW, inverse variance weighted; LD, linkage disequilibrium; LOO analysis, leave‐one‐out analysis; MR‐PRESSO, MR Pleiotropy RESidual Sum and Outlier; SNPs, single nucleotide polymorphisms. This figure is created with FigDraw.

We strictly follow the guidelines of strengthening the reporting of observational studies in epidemiology‐MR (STROBE‐MR) (Skrivankova et al. 2021) (Table S1). The overall research framework and a summary of the research procedures are presented in Figure 1. Single nucleotide polymorphisms (SNPs) from GWAS summary data associated with circulating biomarkers and CSF metabolites were identified as instrumental variables (IVs) to determine the causal effects of these factors on brain malignancy. The MR analysis conducted in this study was rigorously guided by three critical assumptions: (1) the selected SNPs were strongly linked to the exposures of interest; (2) the IVs were independent of potential confounding factors; and (3) the genetic variants used as IVs were not connected to alternative pathways that could influence the outcomes (de Leeuw et al. 2022).

### 2.2 Exposure Data and Outcome Data Selection

We sourced the latest and largest GWAS dataset available. The primary genetic instruments were derived from a recent study (Karjalainen et al. 2024), which analyzed nutritionally annotated circulating metabolic traits using nuclear magnetic resonance spectroscopy in over 136,000 participants from 33 cohorts, mostly Europeans. This study includes 213 lipid‐related factors, 20 non‐lipid traits, and far surpasses previous studies involving 25,000 participants. The GWAS Catalog accession number is GSCT90301941‐GSCT90302173. The valuable data included in this investigation for the CSF metabolites originated from a metabolome‐wide association study (MWAS) performed on 689 participants (Panyard et al. 2021). Among them, 338 CSF metabolites were associated with the genome and the datasets requested from the WADRC (https://www.adrc.wisc.edu/apply‐resources).

The GWAS summary data for malignant neoplasm of the brain were obtained from the FinnGen consortium (Version R11; accessed on: 2024). FinnGen, a large‐scale public–private partnership project in Finland, offers a unique opportunity to study genetic variation and disease trajectories in an isolated population (Kurki et al. 2023). The data contain 1070 cases, from the cancer register (ICD‐O‐3), including malignant neoplasm of the brain located in the cerebrum, frontal lobe, temporal lobe, parietal lobe, occipital lobe, ventricle, cerebellum, brain stem, overlapping brain lesions, and unspecified. Diagnosis criteria were based on ICD‐10 C71 (Becker et al. 2021). It also contains 345,118 controls excluding all cancers, which is the most recent data in all databases to date.

### 2.3 Selection of IVs

To ensure the reliability of IVs representing causal relationships, we undertook a rigorous selection process to identify IVs that meet the three key assumptions of MR analysis. We employed a genome‐wide significance threshold (*p* < 5 × 10^−8^) to identify SNPs with strong associations to exposures (Lin et al. 2023). To maintain the independence of selected SNPs and minimize potential bias, we removed SNPs in linkage disequilibrium (LD) by applying a threshold of *r* ^2^ = 0.001 and a distance of 10,000 kb (Chen et al. 2023). The Steiger test was performed to exclude IVs with a greater effect on the outcome than on the exposure, and SNPs were harmonized between exposure and outcome data to eliminate duplicates and mismatches. Then we assessed the strength of the IVs using an *F*‐test, retaining only those with robust genetic variation (*F* > 10) for further analysis (Burgess and Thompson 2011). Finally, after filtering the data, MR analysis was conducted on metabolic traits with more than two SNPs.

### 2.4 Univariable MR (UVMR) Analysis

Addressing pleiotropy concerns, we utilized four MR methods. The inverse variance weighted (IVW) method was employed as the primary effect estimate, which considers the influence of various confounding factors and provides the most accurate and reliable assessment of the effect (Burgess et al. 2015). We also applied the MR‐Egger, the weighted median, and the weighted mode (Hartwig et al. 2017). The *p*‐value < 0.05 indicated that metabolism subjects affected the outcomes. All *p*‐values from the IVW analyses were adjusted for multiple testing using the Benjamini–Hochberg false discovery rate (FDR) method. An FDR‐adjusted *p*‐value < 0.05 was considered statistically significant. Associations with a nominal *p*‐value < 0.05 but not surviving FDR correction were regarded as suggestive causal associations. Sensitivity analysis is crucial as it investigates heterogeneity and horizontal pleiotropy, which can significantly impact MR estimates. Heterogeneity among IVs was evaluated using Cochran's Q statistic (Burgess et al. 2013) under both the IVW and MR‐Egger models. Horizontal pleiotropy was assessed using the MR‐Egger intercept test, where a non‐zero intercept indicates the presence of directional pleiotropy. In addition, the MR Pleiotropy RESidual Sum and Outlier (MR‐PRESSO) method was applied to detect potential pleiotropic outliers using the global and outlier tests, thereby improving the robustness of causal inference (Verbanck et al. 2018). Besides, we conducted a leave‐one‐out (LOO) analysis (Vehtari et al. 2017) to assess bias in the results by removing each SNP one at a time.

### 2.5 Multivariable MR (MVMR) Analysis

Unlike UVMR, which evaluates the total effect of a single exposure on an outcome, MVMR isolates the independent contribution of each exposure (Sanderson 2021). In this study, we applied MVMR to the identified metabolites to control for their interrelationships. The MVMR‐IVW was used as the primary approach for estimating causal effects, supplemented by MVMR‐Egger and MVMR‐Lasso. Sensitivity analyses were conducted using the Cochran's Q test and MR‐Egger intercept. We expressed MR estimates as odds ratios (ORs) with 95% confidence intervals (CIs), indicating the relative risk of each exposure associated with the outcome. The R software was conducted to integrate exposure and outcome data during the process, followed by statistical analysis.

### 2.6 Metabolic Pathway and Enrichment Analysis

After MR analysis, we extracted the positive results obtained after eliminating sensitivity and searched for the metabolite ID numbers from the Human Metabolome Database (HMDB) and Kyoto Encyclopedia of Genes and Genomes (KEGG). The web‐based software MetaboAnalyst 6.0 (Pang et al. 2024) (https://www.metaboanalyst.ca/MetaboAnalyst/faces/home.xhtml) was used to conduct metabolic pathway analysis for the identified metabolites. Furthermore, we enriched and associated suggestive CSF metabolites as disease signatures using existing library data. Chiplot (https://www.chiplot.online/) was used to visualize the results.

## 3 Results

### 3.1 Single Nucleotide Polymorphisms

As for 233 circulating biomarkers, we successfully extracted a total of 13,360 SNPs. Through rigorous screening of IVs, we identified 12,716 corresponding genetic variants for the metabolites from the malignant neoplasm of the brain. The filtered set comprised SNPs ranging from 3 to 88. The F‐statistics of the selected SNPs ranged from 29.7 to 7610.1, all exceeding the conventional threshold of 10, indicating that the instruments were sufficiently strong.

Similarly, a total of 48,776 SNPs for 338 CSF metabolite traits were acquired. Through rigorous screening of IVs, we identified 47,773 corresponding genetic variants, and the filtered set comprised SNPs ranging from 25 to 545. The F‐statistics of SNPs associated with metabolites range from 16.2 to 904.8. The detailed data of IVs are presented in Tables S2 and S3, and all exposures and outcomes passed the Steiger test.

### 3.2 UVMR Estimates

**FIGURE 2:**
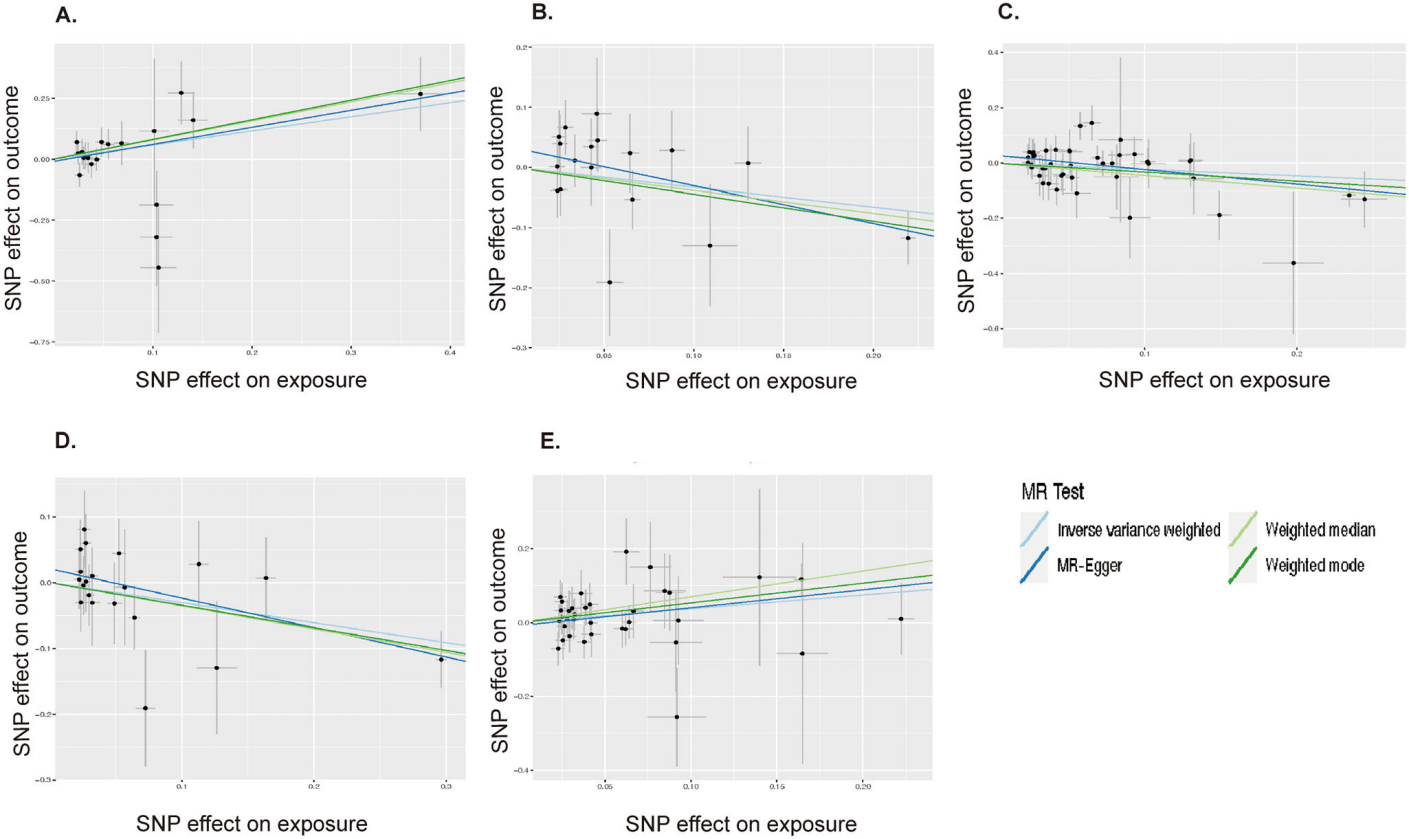
Scatter plot of 5 circulating metabolic biomarkers with a causal relationship with malignant neoplasm of brain across 233 exposures. (A) albumin, (B) ratio of 22:6 docosahexaenoic acid to total fatty acids, (C) omega‐3 fatty acids, (D) ratio of omega‐3 fatty acids to total fatty acids, and (E) ratio of 18:2 linoleic acid to total fatty acids.

The significant effect estimates for associations are presented in Tables S4 and S5. The IVW method, our primary analysis technique, indicated an increased risk of brain malignancy associated with elevated levels of albumin (*p* = 0.03, OR: 1.78, 95% CI: 1.06–3.01) and ratio of 18:2 linoleic acid to total fatty acids (*p* = 0.02, OR: 1.45, 95% CI: 1.06–1.99). By contrast, the levels of omega‐3 fatty acids (*p* = 0.04, OR: 0.79, 95% CI: 0.64–0.99), ratio of 22:6 docosahexaenoic acid (DHA) to total fatty acids (*p* = 0.04, OR: 0.72, 95% CI: 0.52–0.99), and ratio of omega‐3 fatty acids to total fatty acids (*p* = 0.01, OR: 0.74, 95% CI: 0.58–0.94) showed negative correlation with the risk of brain malignancy. These findings were interpreted in the context of multiple testing. The corresponding scatter plot is shown in Figure 2, and all analysis methods showed consistent directionality.

**FIGURE 3:**
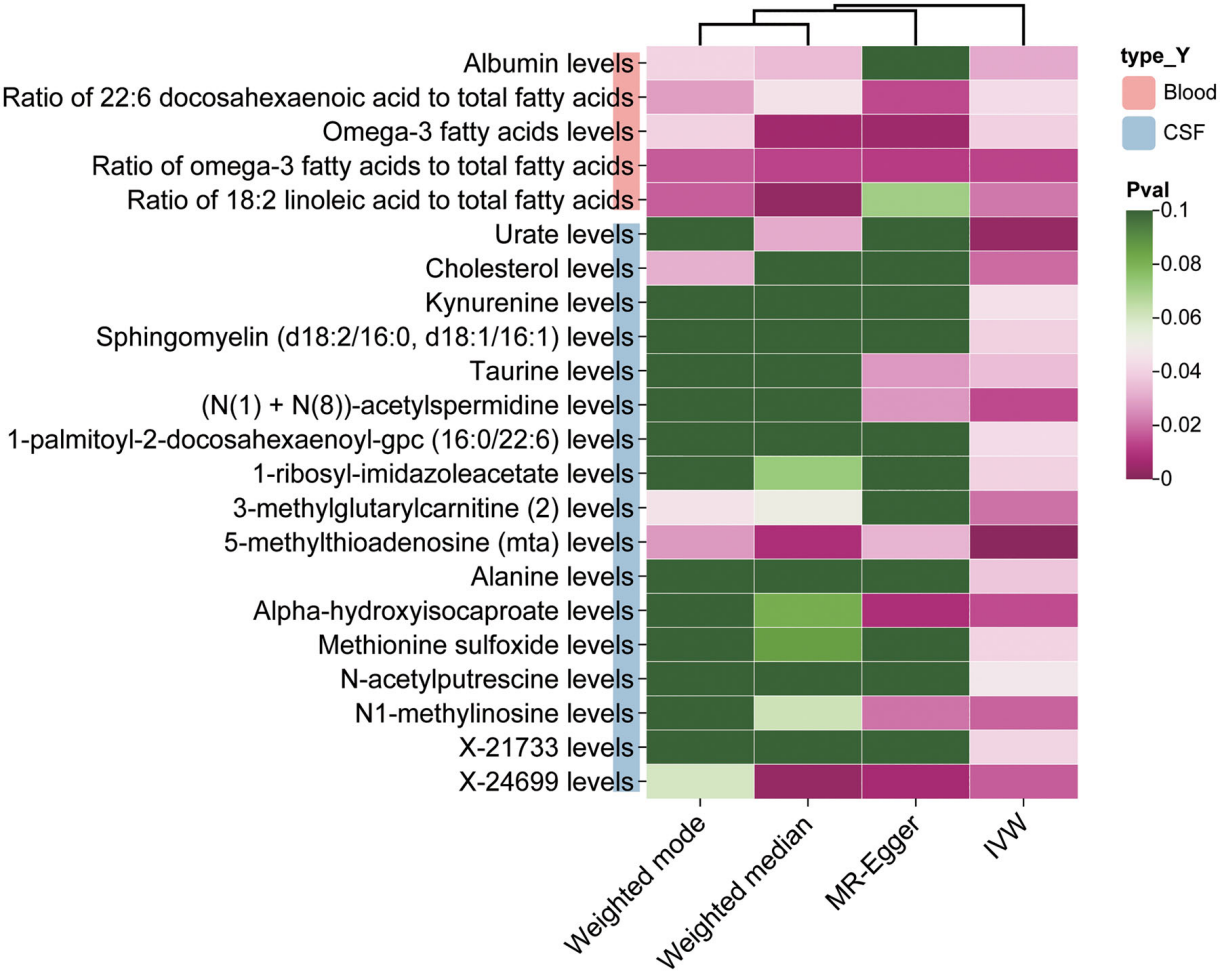
Results of the associations between main metabolites and malignant neoplasm of the brain using four methods.

From CSF metabolite analysis, we found a total of 17 metabolic traits with a causal relationship with malignant neoplasm of the brain, among which there was a negative correlation between urate, cholesterol, kynurenine, (N(1) + N(8))‐acetylspermidine, 3‐methylglutarylcarnitine, N‐acetylputrescine, and N1‐methylinosine levels with the risk of brain malignancy. The levels of 5‐methylthioadenosine (MTA) (*p* = 0.001, OR: 1.25, 95% CI: 1.09–1.44) had the strongest association. The scatter plot is shown in Figure S1, and the heat map displaying the analysis results of the four MR methods is presented in Figure 3.

### 3.3 Sensitivity Analysis

All our primary results passed the heterogeneity test using Cochran's Q test. The MR‐Egger intercept suggested potential pleiotropy for omega‐3 fatty acid levels (*p* = 0.04), whereas no evidence of horizontal pleiotropy was observed for the remaining associations (*p* > 0.05). MR‐PRESSO global and outlier tests did not identify significant pleiotropic outliers for the reported associations, indicating that the causal estimates were not materially influenced by horizontal pleiotropy. The results of the sensitivity analysis are shown in Table 1. LOO analysis further confirmed the absence of horizontal pleiotropy bias in this context. Forest plots, shown in Figures S2 and S3, demonstrated that no single SNP disproportionately influenced our results, further validating the robustness of our findings.

**TABLE 1:** Main UVMR results and sensitivity analysis of the causal relationship between cerebrospinal fluid and blood metabolites and brain malignancies.

		UVMR Analysis		Pleiotropy test				Heterogeneity test	
		IVW		MR‐Egger Intercept			MR‐PRESSO Global Test	IVW	
Metabolites	nSNP	*p*	OR (95% CI)	Intercept	se	*p*	*p*	Q_df	Q_*p*
Circulating biomarkers									
Albumin levels	19	0.030	1.78 (1.06–3.01)	−0.009	0.02	0.69	0.40	18	0.35
Ratio of 22:6 docosahexaenoic acid to total fatty acids	18	0.043	0.72 (0.52–0.99)	0.032	0.02	0.09	0.37	17	0.35
Ratio of omega‐3 fatty acids to total fatty acids	20	0.013	0.74 (0.58–0.94)	0.021	0.01	0.17	0.63	19	0.65
Ratio of 18:2 linoleic acid to total fatty acids	35	0.021	1.45 (1.06–1.99)	−0.009	0.02	0.58	0.70	34	0.75
CSF metabolites									
Urate levels	87	0.003	0.76 (0.64–0.91)	−0.016	0.02	0.38	0.68	86	0.67
Cholesterol levels	205	0.019	0.88 (0.80–0.98)	−0.003	0.01	0.76	0.56	204	0.56
Kynurenine levels	99	0.044	0.84 (0.71–1.00)	−0.012	0.01	0.38	0.32	98	0.31
Sphingomyelin (d18:2/16:0, d18:1/16:1) levels	273	0.039	1.07 (1.00–1.14)	0.010	0.01	0.40	0.40	272	0.39
Taurine levels	32	0.035	1.65 (1.04–2.63)	−0.048	0.03	0.12	0.53	31	0.52
(N(1) + N(8))‐acetylspermidine levels	239	0.014	0.94 (0.89–0.99)	0.014	0.01	0.26	0.50	238	0.50
1‐palmitoyl‐2‐docosahexaenoyl‐gpc (16:0/22:6) levels	276	0.043	1.08 (1.00–1.16)	−0.007	0.01	0.62	0.18	275	0.17
1‐ribosyl‐imidazoleacetate levels	165	0.040	1.11 (1.00–1.22)	0.013	0.01	0.33	0.67	164	0.66
3‐methylglutarylcarnitine (2) levels	170	0.020	0.90 (0.83–0.98)	−0.013	0.01	0.30	0.14	169	0.13
5‐methylthioadenosine (MTA) levels	192	0.001	1.25 (1.09–1.44)	−0.004	0.01	0.76	0.28	191	0.28
Alanine levels	70	0.037	1.39 (1.02–1.90)	−0.002	0.02	0.92	0.64	69	0.64
Alpha‐hydroxyisocaproate levels	90	0.014	1.34 (1.06–1.69)	−0.035	0.02	0.07	0.07	89	0.07
Methionine sulfoxide levels	223	0.040	1.09 (1.00–1.18)	0.007	0.01	0.54	0.29	222	0.27
N‐acetylputrescine levels	95	0.047	0.77 (0.60–1.00)	−0.006	0.02	0.73	0.86	94	0.86
N1‐methylinosine levels	144	0.017	0.83 (0.72–0.97)	0.015	0.01	0.25	0.08	143	0.07
X‐21733 levels	122	0.041	1.05 (1.00–1.10)	−0.001	0.01	0.92	0.23	121	0.23
X‐24699 levels	96	0.017	1.30 (1.05–1.61)	−0.031	0.02	0.06	0.31	95	0.30

### 3.4 MVMR Analysis

After adjusting for metabolite interactions, MVMR estimates using IVW (Table S6) and two complementary methods consistently demonstrated that genetically predicted blood albumin (MVMR‐IVW: *p* = 0.01, OR: 1.90, 95% CI: 1.16–3.12), along with CSF metabolites such as alpha‐hydroxyisocaproate (MVMR‐IVW: *p* = 0.01, OR: 1.60, 95% CI: 1.12–2.29), 1‐ribosyl‐imidazoleacetate (MVMR‐IVW: *p* = 0.04, OR: 1.12, 95% CI: 1.00–1.24), MTA (MVMR‐IVW: *p* = 0.02, OR: 1.24, 95% CI: 1.04–1.47), N1‐methylinosine (MVMR‐IVW: *p* = 4.84 × 10^−4^, OR: 0.75, 95% CI: 0.64–0.88), and urate (MVMR‐IVW: *p* = 4.25 × 10^−3^, OR: 0.74, 95% CI: 0.60–0.91), can directly influence brain malignancy, independent of other metabolites. All findings were robust to sensitivity analyses.

### 3.5 Metabolic Pathway and Enrichment Analysis

**FIGURE 4:**
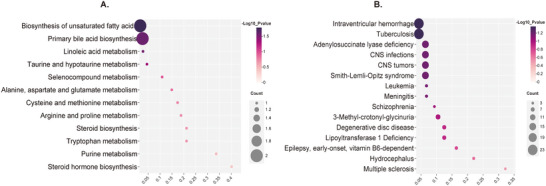
Metabolic pathway enrichment analysis for positive results obtained after eliminating sensitivity. (A) Overview of enriched metabolite sets—Pathway. (B) Overview of enriched metabolite sets (CSF)—Top 15 diseases.

We successfully annotated 12 known metabolites with IDs for 4 blood and 17 CSF metabolites. Our analysis identified 12 metabolic pathways potentially involved in the mechanism of brain malignancy (Figure 4A), with the most notable being biosynthesis of unsaturated fatty acids (*p* = 0.01), primary bile acid biosynthesis (*p* = 0.03), linoleic acid metabolism (*p* = 0.03), and taurine and hypotaurine metabolism (*p* = 0.04). These pathways appear to play significant roles in the biological processes driving brain malignancy. In addition, the top 15 diseases associated with these metabolites were enriched (Figure 4B), with intraventricular hemorrhage and CNS tumors ranking among the best, reinforcing our findings.

## 4 Discussion

Our rigorous MR analysis revealed the causal relationships between circulating metabolic biomarkers, CSF metabolites, and brain malignancy. Overall, 4 blood metabolites and 17 CSF metabolites were potentially associated with the risk of brain malignancy. The results provided fresh insights into the impact of gene–environment interactions on the development of brain malignancy, potentially advancing personalized nutritional intervention strategies in neuro‐oncology.

Several previous studies align with our findings, highlighting key opportunities for preventing and treating. In this study, the ratio of 22:6 DHA to total fatty acids and the ratio of omega‐3 fatty acids to total fatty acids were identified as protective factors. 22:6 DHA, a vital omega‐3 fatty acid in Western diets, supports brain function, vision, and inflammation regulation, earning it the title “brain gold.” Previous studies have suggested that DHA is associated with a reduced risk of brain malignancies, as its metabolites (oxylipins) and FABP‐mediated signaling pathways can suppress glioblastoma progression (Montecillo‐Aguado et al. 2020), enhancing neuroprotective mechanisms and promoting cell death through ferroptosis (Dierge et al. 2021). In contrast, 18:2 linoleic acid, an omega‐6 fatty acid, may promote tumor growth in the brain. High intake of omega‐6 has been linked to increased risks of brain cancers by activating signaling pathways that facilitate tumor progression (Bosch‐Bouju and Layé 2016). Furthermore, elevated levels of *n*‐6 fatty acids can impair mitochondrial function by inhibiting calcium transport, disrupting energy metabolism essential for healthy brain cells (Peppone et al. 2019). This interplay highlights the need for a balanced intake of omega‐3 and omega‐6 fatty acids to mitigate the risk of brain malignancies and support overall brain health.

For CSF metabolites, prior studies suggest their potential as biomarkers. A metabolomics study found that changes in the levels of carbohydrate metabolites such as glucose and lactate in CSF metabolites were closely related to the occurrence and development of primary brain tumors (Baranovičová et al. 2019). Genetic alterations, particularly IDH mutations, have been shown to influence tumor cell metabolic profiles, especially in glycolytic pathways (N. Wang et al. 2024). In our investigation, specific metabolites—uric acid, alanine, and α‐hydroxyisocaproic acid—demonstrated functional linkages to carbohydrate metabolism. Their dynamic changes indicate indirect glycolytic dysregulation and may drive tumor microenvironment remodeling via immunomodulatory mechanisms. In addition, methionine sulfoxide is linked to oxidative stress, reflecting the antioxidant status of tumor cells. Kynurenine levels may signal tumor‐related immunosuppression and change in the microenvironment, while (N(1) + N(8)) acetylspermidine affects cell proliferation and apoptosis (Kawakita and Hiramatsu 2006). 3‐Methylglutarylcarnitine levels are associated with fatty acid oxidation and mitochondrial metabolism.

The connection between MTA and brain malignancy is notably significant. Elevated MTA levels in our study may indicate a strong causal relationship with brain tumor development. Previous work has shown that MTA accumulates in various cancers, plays a key role in tumor metabolism, and may regulate tumors by modulating the Akt signal pathway (Henrich et al. 2016). MTA suppresses T cell and NK cell function, facilitating tumor immune escape. Clinically, dynamic MTA fluctuations exhibit temporal correlation with glioblastoma progression metrics, positioning it as a dual‐purpose biomarker for disease monitoring and immunotherapy response prediction (Brummer et al. 2024). Therapeutically, pharmacological targeting of MTA biosynthesis pathways represents a promising strategy to reverse tumor‐mediated immune paralysis (Briggs et al. 2025).

Our metabolic pathway analysis also revealed several notable findings. Taurine and hypotaurine metabolism exhibit a significant association with brain malignancy. Taurine has been identified as an important metabolite for detecting human breast cancer based on the targeted metabolomics analysis (X. Wang et al. 2018). Hypotaurine, a precursor of taurine, is markedly elevated in brain tumors and has been shown to promote glioma stem cell self‐renewal and maintenance by activating the NF‐κB pathway, thereby driving tumor growth and progression (Shen et al. 2021). This aligns with our finding that elevated taurine levels act as a promoting factor in tumor development. The role of primary bile acids in tumor cells remains unclear, but studies suggest that alterations in bile acid metabolism may contribute to tumor development (Varanasi et al. 2025). As signaling molecules, bile acids can influence tumor cell proliferation and apoptosis. A clinical research study showed that the bile acid profile was associated with CSF biomarkers in some brain diseases, such as Alzheimer's disease (Nabizadeh et al. 2024).

Our study offers several key advantages. This study represents an initial attempt to methodically evaluate the causal relationship between circulating and CSF metabolites in brain malignancy with MR. The selected SNPs are robust, peer‐reviewed, and sourced from the largest available samples. We employed validated methods to establish the causal relationship between metabolites and brain malignancy, conducting dual‐strength analyses of MVMR and extensive metabolic pathway assessments.

However, due to data limitations, the study is confined to participants of European ancestry and patients with brain malignancies, which may affect its generalizability. While we utilized the most recent and largest brain tumor samples, further large‐scale studies are essential to improve accuracy. We preliminarily investigated whether the biomarkers identified in blood exhibit MR signals in CSF, but no significant correlation was found. This may be due to the dynamic variations of metabolites in the two tissues and the selective permeability of the blood–brain barrier.

## 5 Conclusion

Using complementary approaches from human genomic and metabolomic data, we were able to identify human metabolites that may influence the development and progression of brain malignancy. In summary, our study identified 4 blood metabolites and 17 CSF metabolites likely linked to the risk of brain malignancy. In addition, 12 metabolic pathways were annotated as related to brain malignancy, with 4 showing significant correlations. The discovery of these metabolites offers valuable insights for early prevention and treatment, and can serve as a foundation for understanding other cancers. Furthermore, it facilitates the integration of translational and clinical research to explain how these metabolites induce the development and progression of brain tumors.

## Author Contributions

**Xinyin Zhang**: investigation, methodology, project administration, visualization, writing – original draft. **Zhicheng Wu**: data curation, review. **Jing Tan**: methodology, data curation. **Mengting Hu**: visualization, validation. **Feixia Pan**: funding acquisition, conceptualization, supervision, writing – review and editing. **Ting Tao**: funding acquisition, conceptualization, supervision, writing – review and editing. All authors contributed to the planning, execution, and analysis of the study and approved the final submitted version.

## Funding

This study was supported by a grant (No. 2024C03181) from the Key R&D Program of Zhejiang Province, a grant (No. X2024174) from the Student Research and Training Program of Zhejiang University, and a startup fund from the Children's Hospital, Zhejiang University School of Medicine.

## Ethics Statement

All data in this study are available in publicly available databases. No additional ethical approval was required.

## Conflicts of Interest

The authors declare no conflicts of interest.

## Supporting information




**Supplementary Materials**: brb371319‐sup‐0001‐figuresS1‐S3.docx

**Supplementary Materials**: brb371319‐sup‐0002‐ tablesS1‐S6.xlsx

## Data Availability

We have marked the sources of all original data in the article.
