# Supplementary material for: Causal Associations of Cerebrospinal Fluid and Circulating Metabolites With Malignant Brain Neoplasms: A Mendelian Randomization Analysis
Source: Brain Behav. 2026 Mar 31;16(4):e71319. doi: 10.1002/brb3.71319 (PMC13111991; doi:10.1002/brb3.71319)

**Supplementary Materials**

**Titles of all supplementary tables:**

**Table S1.** STROBE-MR checklist of recommended items to address in reports of Mendelian randomization studies.

**Table S2.** Characteristics of the genetic variants associated with the 233 circulating biomarkers.

**Table S3.** Characteristics of the genetic variants associated with the 338 CSF metabolites.

**Table S4.** Effect estimates for associations between blood metabolite levels and risk of brain malignancy.

**Table S5.** Effect estimates for associations between CSF metabolite levels and risk of brain malignancy.

**Table S6.** Positive MVMR results and sensitivity analysis of the causal relationship between cerebrospinal fluid and blood metabolites and brain malignancies.

**(Note: We packaged and uploaded these tables in EXCEL format named: Supplement_Table.xlsx)**

**Legends of all supplementary figures:**

**Figure S1.** Scatter plot of 17 CSF metabolic biomarkers with a causal relationship with malignant neoplasm of brain.

**Figure S2.** LOO sensitivity analysis of 4 blood metabolic biomarkers on malignant neoplasm of brain.

**Figure S3.** LOO sensitivity analysis of 17 CSF metabolic biomarkers on malignant neoplasm of brain.

**Figure S1.** Scatter plot of 17 CSF metabolic biomarkers with a causal relationship with malignant neoplasm of brain.


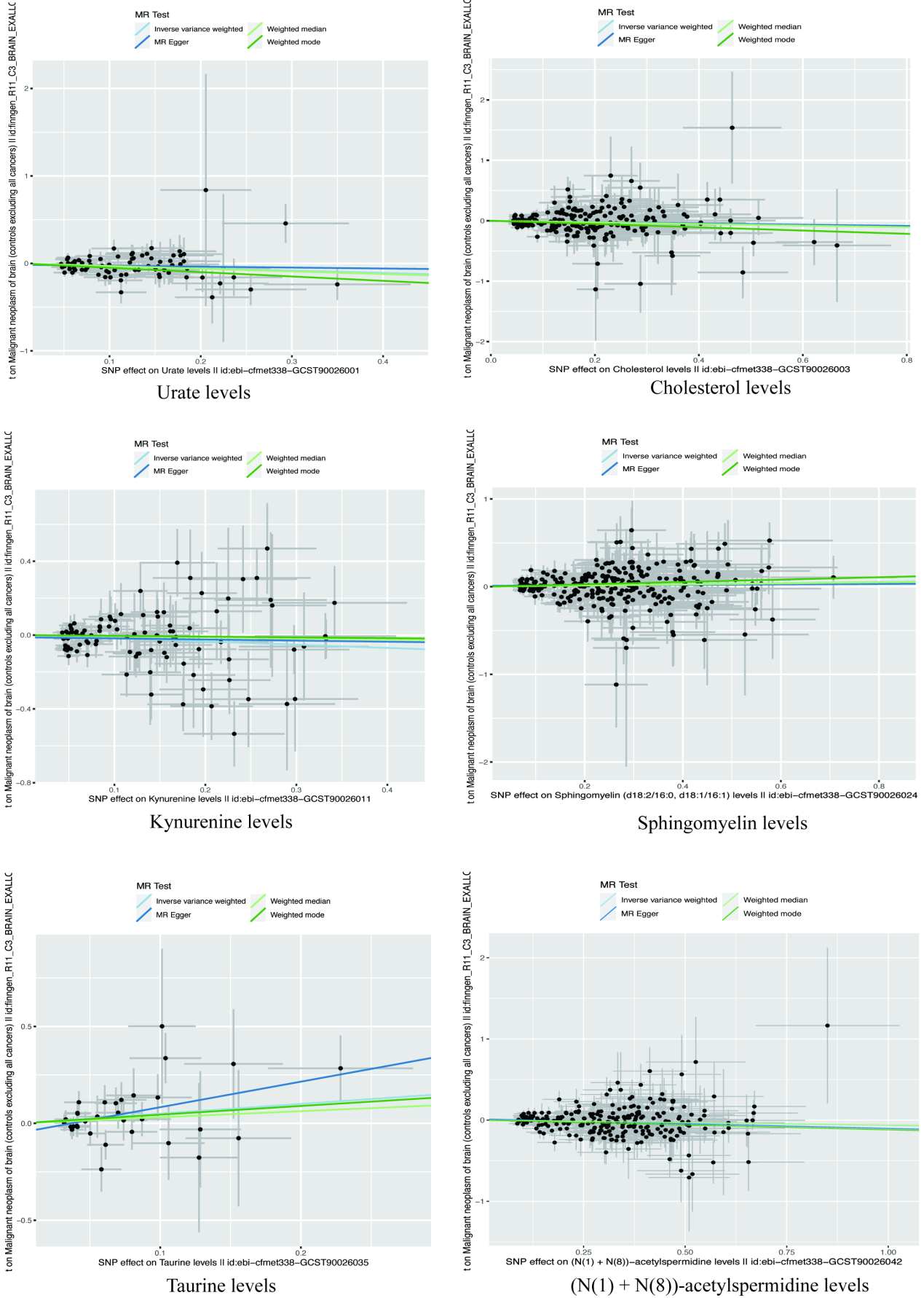


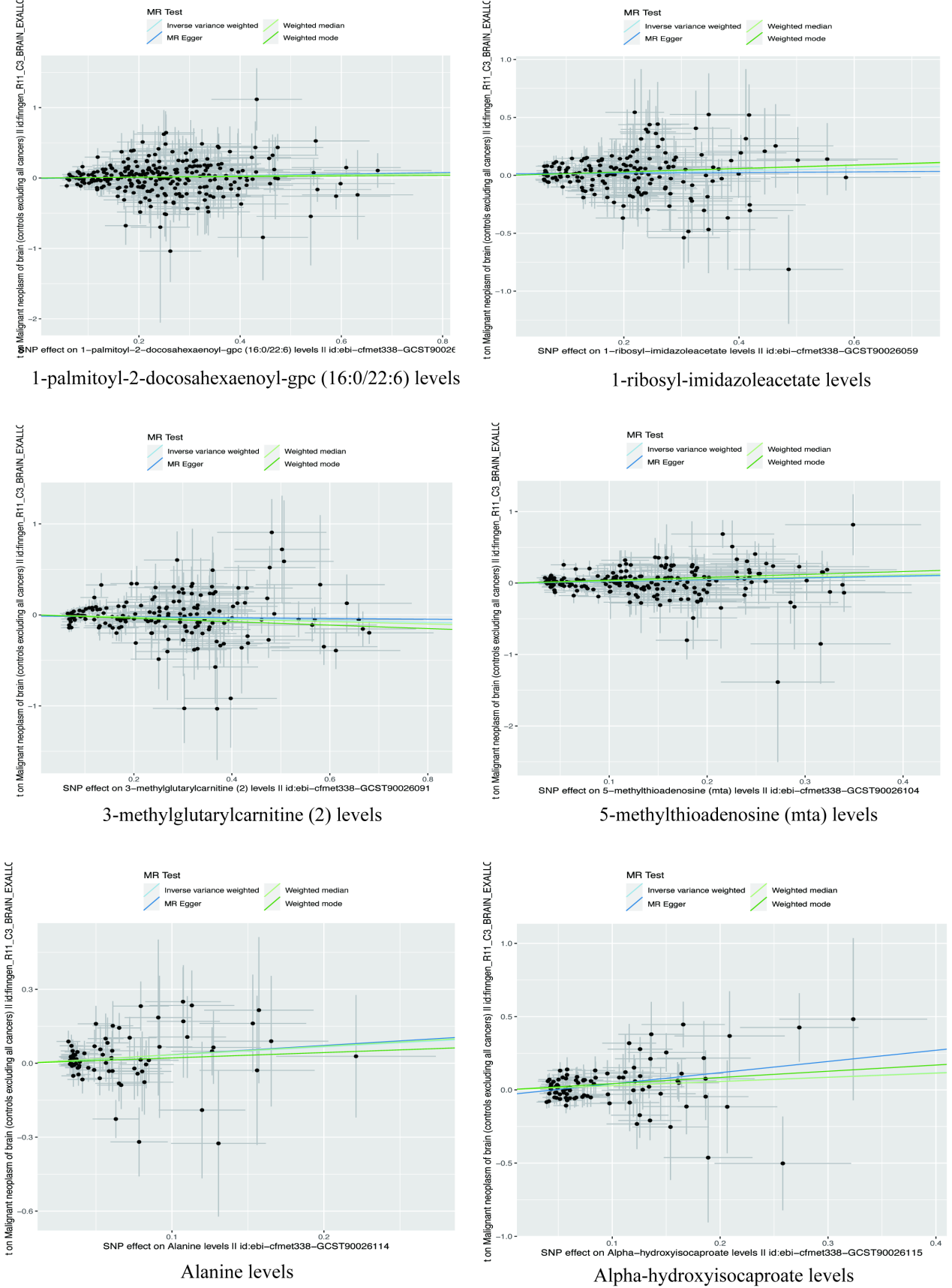


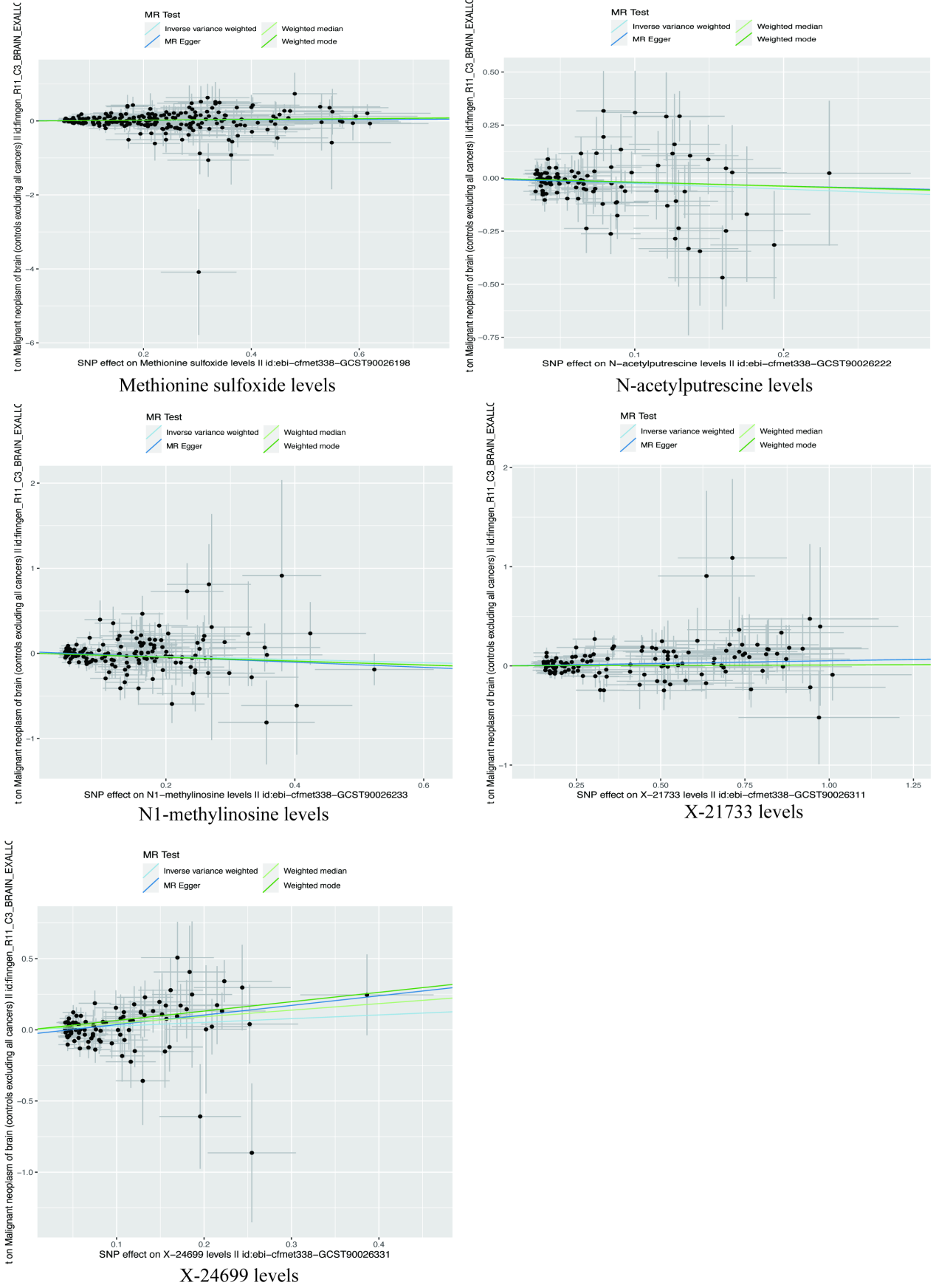


**Figure S2.** LOO sensitivity analysis of 4 blood metabolic biomarkers on malignant neoplasm of brain.


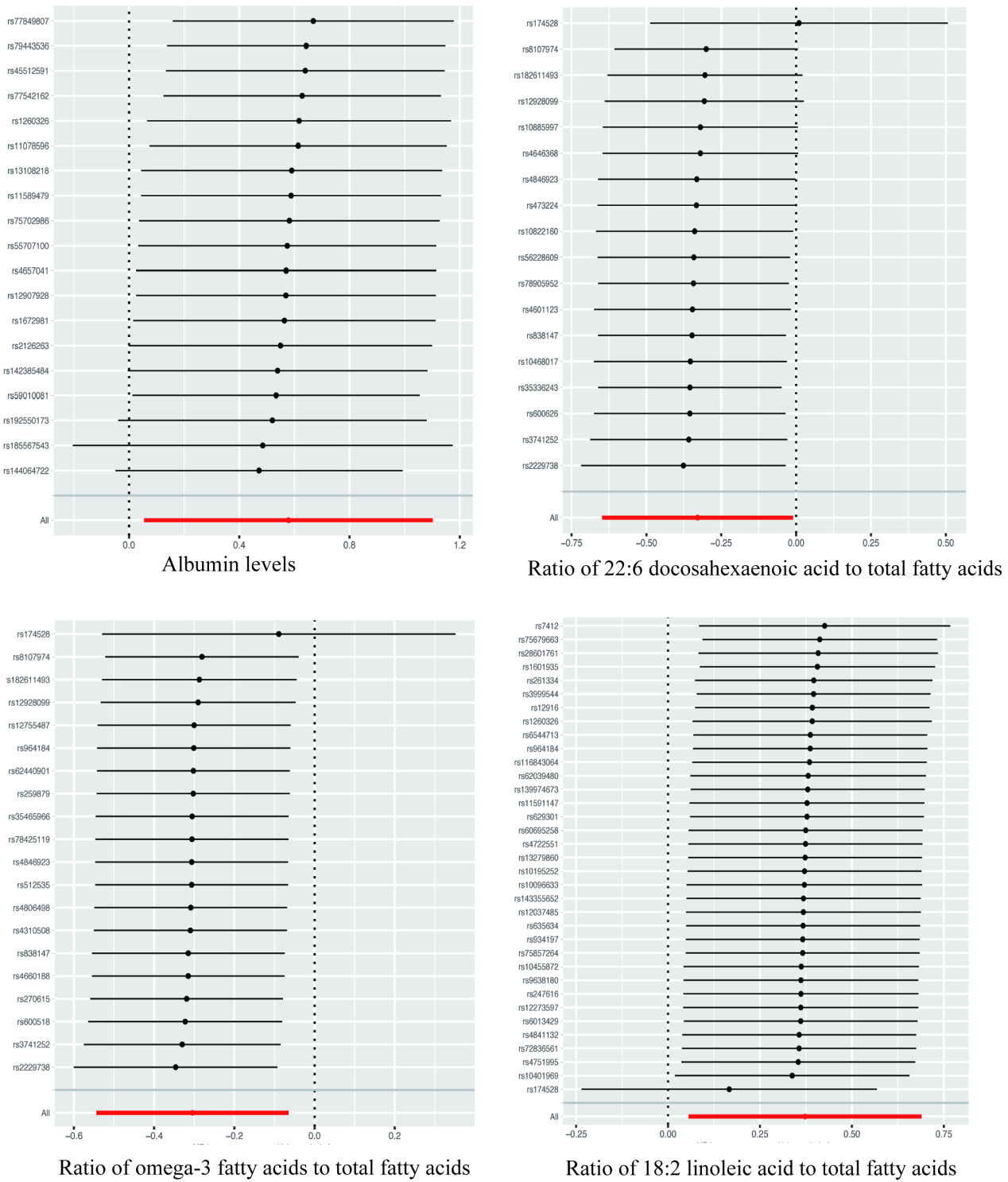


**
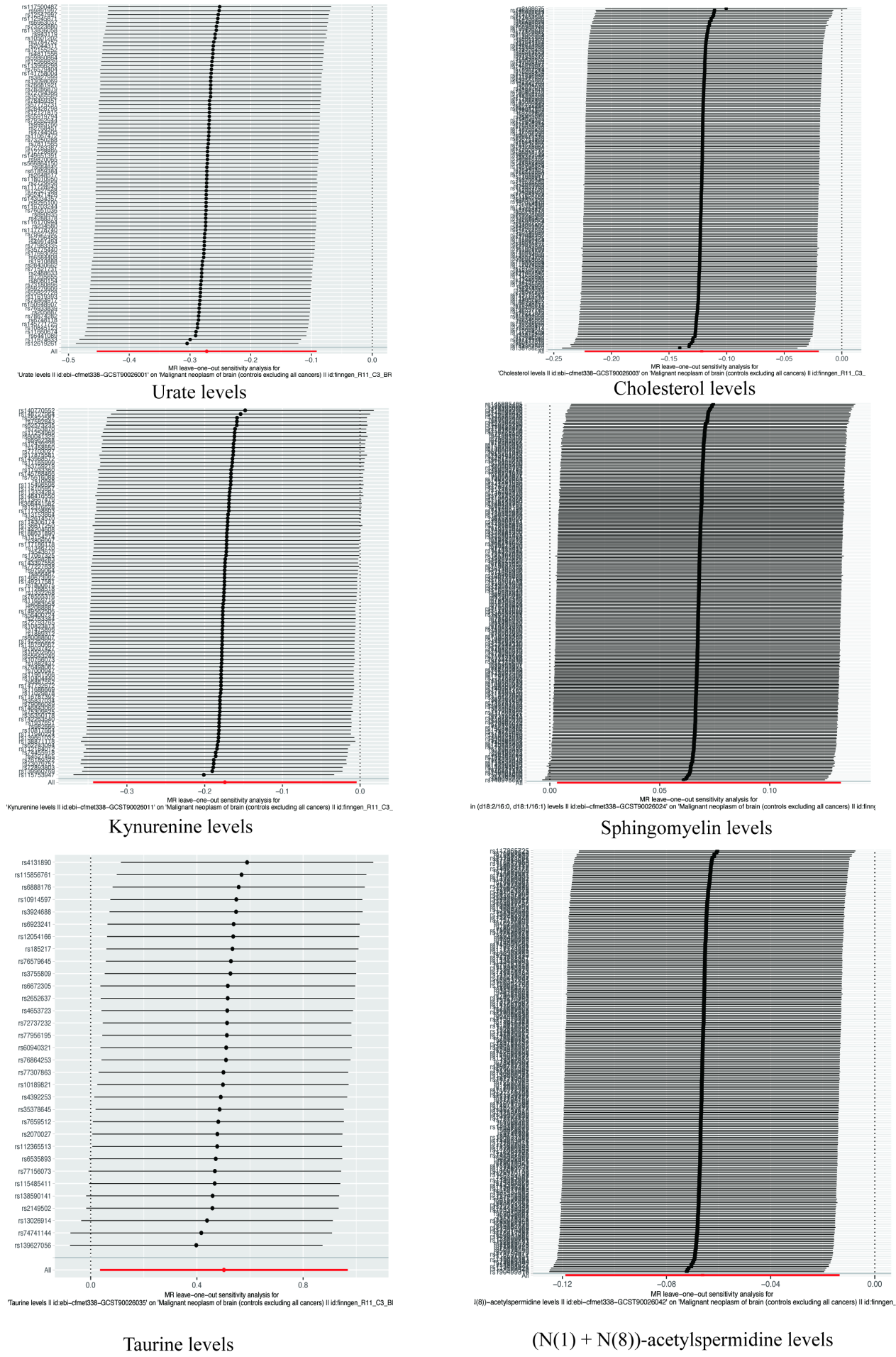
Figure S3.** LOO sensitivity analysis of 17 CSF metabolic biomarkers on malignant neoplasm of brain.


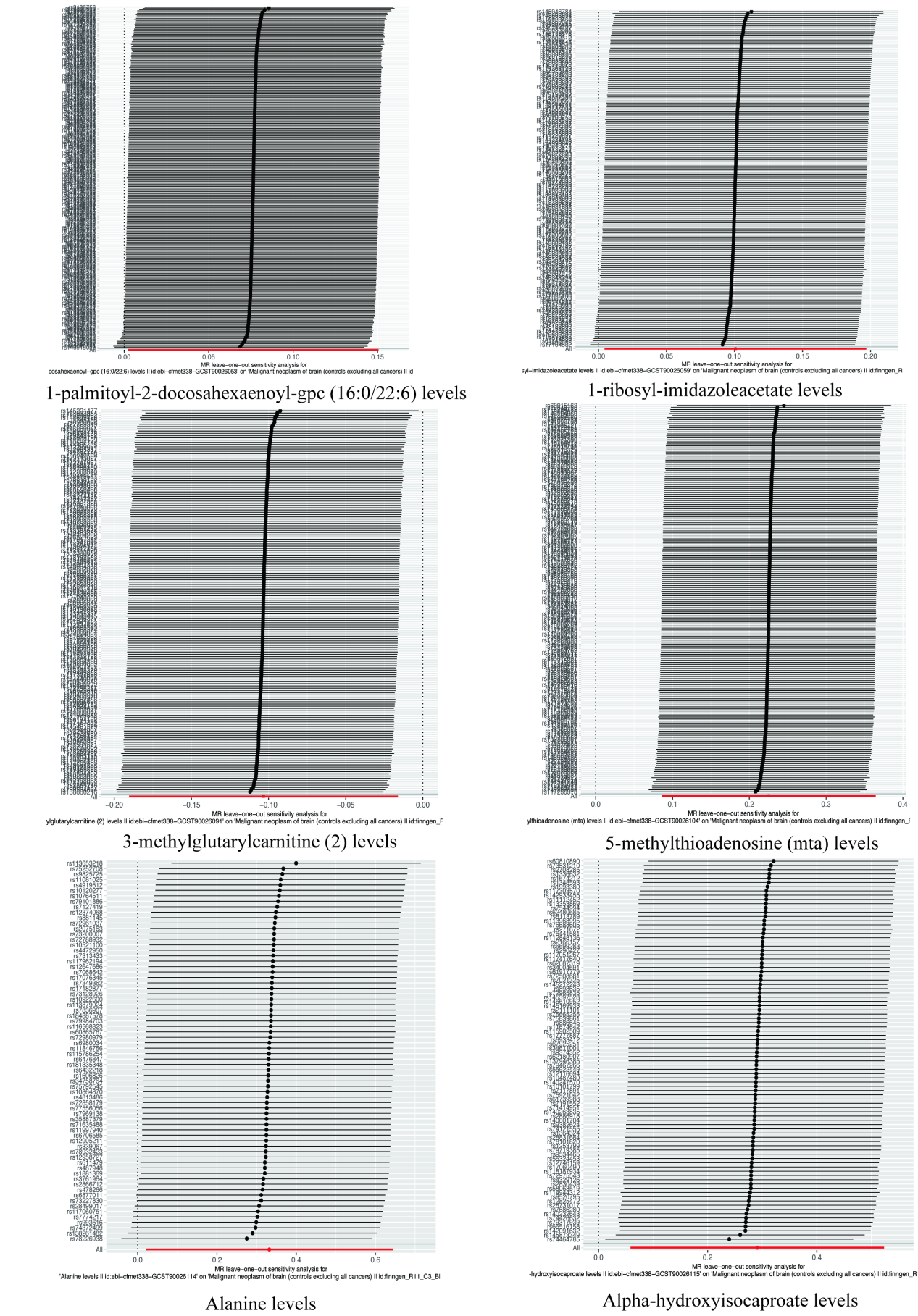


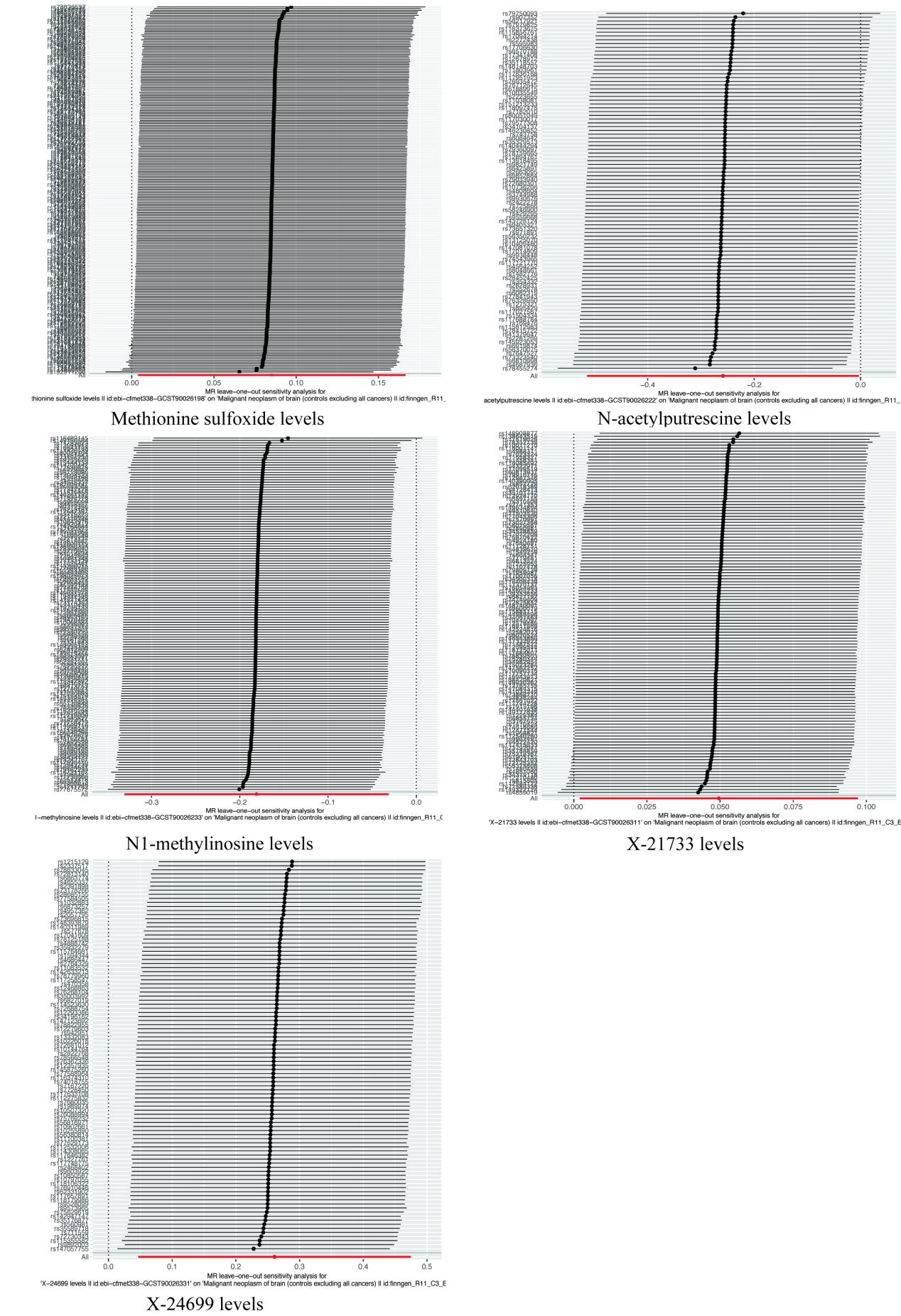

Supplement: Supplementary file 1 — Supplementary Materials: brb371319‐sup‐0001‐figuresS1‐S3.docx [file BRB3-16-e71319-s001.docx]
